# Les effets bénéfiques de l'accompagnement du patient cancéreux: particularités du Maroc

**DOI:** 10.11604/pamj.2014.19.93.4533

**Published:** 2014-09-26

**Authors:** Sihame Lkhoyaali, Meryem Aitelhaj, Hassan Errihani

**Affiliations:** 1 Service d'Oncologie Médicale, Institut National d'Oncologie, Rabat, Maroc

**Keywords:** Patient cancéreux, Maroc, surestime, cancer patient, Morocco, overestimate

## Abstract

Au Maroc la majorité des patients âgés cancéreux sont pris en charge par leurs proches l'accompagnement des patients est à l'origine de conséquences émotionnelles psychiques et financières négatives mais en contrepartie il est à l'origine de plusieurs effets bénéfiques à savoir un resserrement des liens familiaux, une surestime du soi et il est la source d'un bien-être affectif et spirituel qui permet de faire face à la maladie.

## Aux éditeurs du Journal Panafricain de Médecine

La place des proches considérés par les professionnels de santé comme les aidants naturels, dans la prise en charge des patients cancéreux a pris un intérêt majeur ces dernières années. Cet intérêt s'inscrit dans un consensus concernant l'avantage du maintien des personnes atteintes de cancer dans leur milieu naturel, mais aussi dans un climat d'inquiétude quant au vieillissement de la population et ses conséquences sur les coûts des services socio-sanitaires.

L'accompagnement d'un patient cancéreux n'est pas une simple tâche, il constitue une expérience douloureuse qui finit le plus souvent par la perte d'un être très cher avec tout ce qui en découle de stress et douleur morale, et peut avoir des conséquences émotionnelles et physiques majeures chez les proches. Ces derniers ne sont généralement pas préparés à ce défi, ils assument leurs tâches pour des raisons qui incluent le sentiment d′obligation envers leur proche et en plus des raisons plus pratiques qui sont le manque de moyens et de personnel soignant [1].

La motivation de cet accompagnement par les membres de la famille est essentiellement volontaire, liée aux obligations de l′attachement entre les membres de la famille, peut également être affectée par les normes culturelles sociales et surtout religieuses, mais aussi peut être liée aux sentiments de culpabilité envers les parents [2]. Les patients âgés ont des besoins spéciaux ils souffrent beaucoup plus d'impotence fonctionnelle, ont moins de capacités cognitives, leurs proches constitués essentiellement par leurs enfants et leurs conjointes ont pour rôle de les accompagner pendant leurs visites fréquentes au centre de traitement et de faire face aux effets secondaires des médicaments et aider aux activités de la vie quotidienne [3].

Au Maroc, la famille joue un rôle majeur dans les soins des patients âgés atteints de cancer. La majorité des patients âgés vivent avec leurs familles qui fournissent l′aide financière psychologique et physique ainsi que les soins requis. Plusieurs études indiquent que les patients afro-américains sont plus susceptibles d′être pris en charge par un membre de leur famille (essentiellement les enfants adultes) par rapport aux caucasiens [4]. Cette particularité ethnique au Maroc et dans plusieurs pays africains est souvent soulignée dans notre pratique courante; la famille est une source de soutien physique, psychique et financier inépuisable.

Plusieurs études ont démontré que la participation de la famille dans la prise en charge des patients atteints de maladies chroniques facilite l'acceptation de la maladie [5] et permet l'adhérence au traitement, [6]. L'accompagnement et l'assistance du patient est à la fois une tache difficile mais reste une expérience enrichissante. Une source de gratification et d’épanouissement pour l'aidant naturel [7]. Puisque la majorité des proches rapportent des effets bénéfiques de l'accompagnement, à noter un resserrement des liens familiaux, une valorisation du temps passé avec le patient, et une occasion d'apprentissages nouveaux [8]. D'autres effets positifs ont été relevés concernant la capacité d'ajustement positif aux événements, l'amélioration des relations interpersonnelles, le développement de l'empathie, la croissance spirituelle et émotionnelle, la surestime de soi. Des études ont montré que le fait de prendre soin d′un patient atteint de cancer donne un sentiment de satisfaction, permet le rapprochement avec le patient, et un sentiment de s′acquitter d′une obligation. Les aspects positifs de l'accompagnement sont associés au bien-être psychologique et la volonté de l′aidant à continuer de fournir des soins [9].

## Conclusion

Au Maroc la majorité des patients âgés cancéreux sont pris en charge par leurs proches constitués essentiellement par leurs enfants et leurs conjoints, l'accompagnement des patients est à l'origine de conséquences émotionnelles psychiques et financières négatives mais en contrepartie il permet un resserrement des liens familiaux, une surestime du soi et il est à l'origine d'un bien-être affectif et spirituel qui permet de faire face à la maladie.
